# HIF-1α-activated long non-coding RNA KDM4A-AS1 promotes hepatocellular carcinoma progression via the miR-411-5p/KPNA2/AKT pathway

**DOI:** 10.1038/s41419-021-04449-2

**Published:** 2021-12-13

**Authors:** Tianxiang Chen, Runkun Liu, Yongshen Niu, Huanye Mo, Hao Wang, Ye Lu, Liang Wang, Liankang Sun, Yufeng Wang, Kangsheng Tu, Qingguang Liu

**Affiliations:** Department of Hepatobiliary Surgery, The First Affiliated Hospital of Xi’an Jiaotong University, 710061 Xi’an, China

**Keywords:** Liver cancer, Long non-coding RNAs, Prognostic markers, Liver cancer

## Abstract

Hepatocellular carcinoma (HCC) is the most common type of liver cancer with poor clinical outcomes. Long non-coding RNAs (lncRNAs) are extensively involved in the tumorigenesis and progression of HCC. However, more investigations should be carried out on novel lncRNAs and their effects on HCC. Here we identified a novel lncRNA KDM4A-AS1, which was aberrantly overexpressed in HCC tissues, associated with unfavorable clinical features and poor prognosis of patients. KDM4A-AS1 promoted HCC cell proliferation, migration, and invasion in vitro and contributed to HCC growth and lung metastasis in vivo. Mechanistically, KDM4A-AS1 was inversely modulated by miR-411-5p at the post-transcriptional level and facilitated Karyopherin α2 (KPNA2) expression by competitively binding miR-411-5p, thereby activating the AKT pathway. KPNA2 silencing, miR-411-5p overexpression, and AKT inhibitor (MK2206) consistently reversed KDM4A-AS1-enhanced proliferation, mobility, and EMT of HCC cells. KDM4A-AS1 was identified as a novel hypoxia-responsive gene and transactivated by hypoxia-inducible factor 1α (HIF-1α) in HCC cells. In turn, KDM4A-AS1 regulated HIF-1α expression through the KPNA2/AKT signaling pathway. Hence, this study revealed a novel hypoxia-responsive lncRNA, KDM4A-AS1, which contributed to HCC growth and metastasis via the KDM4A-AS1/KPNA2/HIF-1α signaling loop. Our findings provide a promising prognostic and therapeutic target for HCC.

## Introduction

Hepatocellular carcinoma (HCC) is the most common liver malignancy and the fourth leading cause of cancer mortality worldwide [1]. Although tremendous efforts have been made to find novel diagnostic and therapeutic targets in the past few decades, the treatment effect of HCC is still unsatisfactory [2]. Due to the insidious onset and rapid progress of HCC, many patients are diagnosed at an advanced stage and not suitable to receive curative treatment [3]. Therefore, it is essential to search for more efficient diagnosis and treatment methods targeting the intrinsic pivotal signaling pathways in HCC.

Long non-coding RNAs (lncRNAs) are a class of RNAs with a minimum length of 200 nucleotides and limited protein-coding potential [4]. Many studies have confirmed that cytoplasm lncRNAs can function as competing endogenous RNAs (ceRNAs) by regulating target genes via direct competition for microRNA (miRNA) binding via sharing miRNA-targeting sites with mRNA [5]. Accumulating evidence has demonstrated that lncRNAs play a remarkable role in the progression of various malignancies, such as HCC [6]. For instance, Geng Qin et al. found that p53-stabilizing and activating RNA inhibits HCC cell proliferation and tumorigenicity via enhancing the accumulation and transactivation of p53 [7]. Hu et al. reported that lincSCRG1 sponges miR-26a to upregulate S phase kinase-related protein 2, thereby promoting the proliferation and migration of HCC cells [8]. Our previous study shows that MCM3AP-AS1 acts as a ceRNA of miR-194-5p to restrain the depression of forkhead box A1, thereby promoting HCC tumor growth [9]. Moreover, we discover that AGAP2-AS1 exerts an oncogenic role in HCC via miR-16-5p/ANXA11 signaling pathway [10]. In addition, we disclose the critical roles of CASC2, RUNX1-IT1, DSCR8, and MAPKAPK5-AS1 in HCC [11–14]. However, it remains tremendously obscure regarding the biological functions and the molecular mechanisms of various novel lncRNAs in HCC.

Increasing amounts of evidence demonstrated that hypoxia is present ubiquitously in solid tumors and plays a crucial role in various biological processes in kinds of malignancies, including HCC [15]. Hypoxia-inducible factor 1α (HIF-1α), accumulated in the hypoxic microenvironment, functions as a transcription factor to regulate the target gene expression [16]. Moreover, recent studies revealed that hypoxia is involved in modulating lncRNAs [12, 14, 17, 18]. For example, hypoxia-induced LUCAT1 contributes to colorectal cancer cell proliferation and chemoresistance via interacting with polypyrimidine tract binding protein 1 (PTBP1) [17]. Hypoxia-responsive BCRT1 is involved in breast cancer progression via the miR-1303/PTBP3 pathway [18]. Our previous study finds that MAPKAPK5-AS1 is induced by hypoxia and mediates HCC progression via miR-154-5p/PLAGL2/HIF-1α signaling loop [14]. RUNX1-IT1 is repressed by hypoxia-driven histone deacetylase 3 and inhibits cell proliferation and cancer stem-like properties in HCC cells [12]. Nevertheless, the explicit relationships between hypoxia and lncRNA remain largely mysterious. It is imperative to conduct further investigations to determine hypoxia-responsive lncRNAs and their biological functions in HCC.

Herein we identified a novel hypoxia-responsive lncRNA, KDM4A-AS1, upregulated in HCC within The Cancer Genome Atlas (TCGA) and Gene Expression Omnibus (GEO) datasets. Furthermore, we explored the expression, clinical significance, and biological function of KDM4A-AS1. Moreover, we investigated the mechanisms underlying the role of KDM4A-AS1 in HCC. Finally, we determined the regulatory mechanism involved in hypoxia-induced KDM4A-AS1 in HCC cells.

## Results

### KDM4A-AS1 is overexpressed in HCC and correlated with poor prognosis

**Fig. 1:**
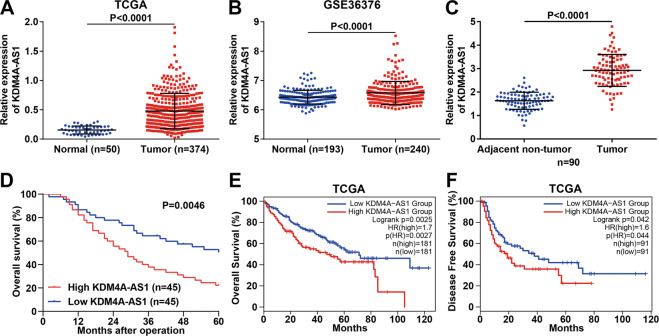
KDM4A-AS1 is upregulated in HCC and associated with a poor prognosis. **A** The expression of KDM4A-AS1 in HCC and normal liver tissues from the TCGA database. **B** KDM4A-AS1 expression in HCC and normal liver tissues from the GSE36376 dataset. **C** RT-qPCR was performed to determine the KDM4A-AS1 level in 90 pairs of HCC and corresponding adjacent non-tumor tissues. **D** Kaplan–Meier analysis was utilized to analyze the overall survival of HCC patients with high or low KDM4A-AS1 expression. **E** The overall survival (OS) and **F** disease-free survival (DFS) of HCC patients with high or low KDM4A-AS1 expression in the TCGA database. All the data are presented as mean ± SD.

We first unraveled dysregulated lncRNAs in HCC using TCGA and GEO databases. KDM4A-AS1, which was significantly upregulated in HCC tissues compared to normal liver tissues in both the TCGA database (*P* < 0.0001, Fig. 1A) and GSE36376 dataset (*P* < 0.0001, Fig. 1B), caught our attention. Furthermore, reverse transcription–quantitative polymerase chain reaction (RT-qPCR) analysis in 90 pairs of HCC and adjacent non-tumor tissues showed that KDM4A-AS1 was highly expressed in HCC tissues compared to non-tumor tissues (*P* < 0.0001, Fig. 1C). Consistently, KDM4A-AS1 was also overexpressed in HCC cell lines (Hep3B, Huh7, HepG2, HCCLM3, MHCC-97H, SK-Hep-1) compared to normal liver cells MIHA (*P* < 0.05, respectively, Supplementary Fig. 1). Then, we divided HCC patients into two subgroups (low/high KDM4A-AS1 group) according to the median of KDM4A-AS1 expression in HCC tissues. As shown in Supplementary Table 1, high KDM4A-AS1 expression was significantly associated with large tumor size (*P* = 0.003), venous infiltration (*P* = 0.011), and advanced tumor, node, metastasis stage (*P* = 0.009). Kaplan–Meier survival analysis revealed that HCC patients with high KDM4A-AS1 expression underwent unfavorable overall survival (OS; *P* = 0.0046, Fig. 1D). TCGA data from GEPIA2 [19] also confirmed that high KDM4A-AS1 expression was relevant to worse OS (*P* = 0.0025, Fig. 1E) and disease-free survival (*P* = 0.042, Fig. 1F) of HCC patients. Thus, we demonstrated that KDM4A-AS1 was overexpressed in HCC, correlated with unfavorable prognosis of HCC patients.

### KDM4A-AS1 acts as an oncogene in HCC

**Fig. 2:**
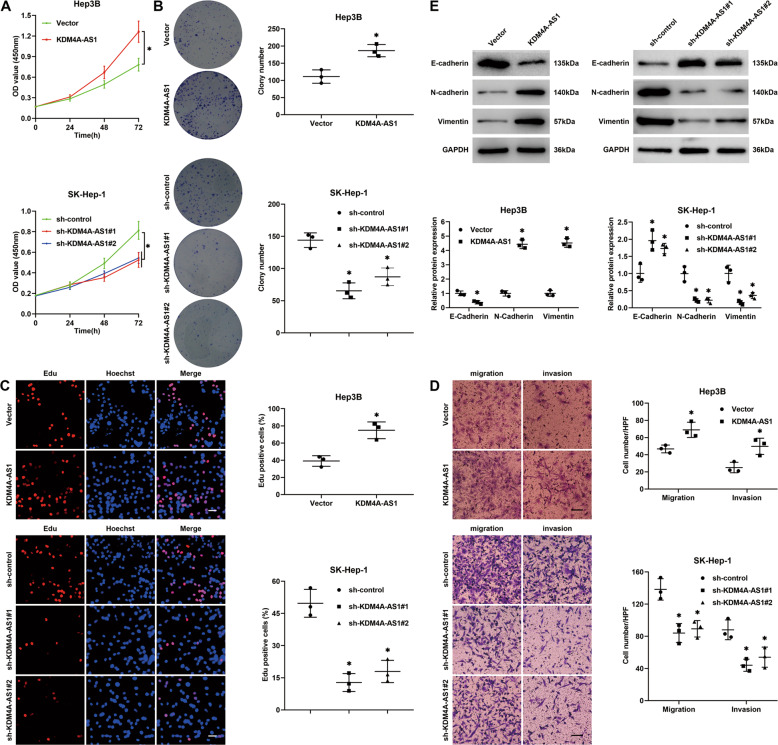
KDM4A-AS1 promotes HCC cell proliferation, migration, and invasion. **A** Hep3B cells were transfected with a vector carrying KDM4A-AS1 or empty vector, and KDM4A-AS1 shRNA (sh-KDM4A-AS1#1 and sh-KDM4A-AS1#2) or nontargeting shRNA (sh-control) was transfected into SK-Hep-1 cells. CCK-8 assay was performed to determine the effect of KDM4A-AS1 overexpression or knockdown on HCC cell viability. **B** Colony formation assay was utilized to evaluate the function of KDM4A-AS1 in HCC cell proliferation. **C** Edu assay was carried out to explore the proliferation of HCC cells affected by KDM4A-AS1 overexpression or knockdown. Scale bar: 20 μm. **D** The effect of KDM4A-AS1 on the migration and invasion abilities of HCC cells was detected by the transwell assay. Scale bar: 100 μm. **E** E-cadherin, N-cadherin, and Vimentin levels were detected to identify the EMT process of HCC cells affected by KDM4A-AS1 overexpression or knockdown. All the data are presented as mean ± SD of at least three independent experiments. *P < 0.05.

Vector carrying KDM4A-AS1 or empty vector was transfected into Hep3B and Huh7 cells, which exhibited low endogenous KDM4A-AS1 level, while KDM4A-AS1 short hairpin RNA (shRNA) and scrambled shRNA were, respectively, transfected into SK-Hep-1 and MHCC-97H cells, which showed a high endogenous KDM4A-AS1 level. RT-qPCR confirmed the satisfactory transfection efficiency (*P* < 0.05, Supplementary Fig. 2). Cell Counting Kit-8 (CCK-8), colony formation, and 5-ethynyl-2′-deoxyuridine (Edu) assays showed that KDM4A-AS1 overexpression notably enhanced HCC cell growth (*P* < 0.05, Fig. 2A–C and Supplementary Fig. 3A–C), while KDM4A-AS1 knockdown remarkably restrained SK-Hep-1 and MHCC-97H cell proliferation (*P* < 0.05, Fig. 2A–C and Supplementary Fig. 3A–C). In addition, transwell assays revealed that KDM4A-AS1-overexpressing Hep3B and Huh7 cells exhibited drastically strengthened migration and invasion abilities (*P* < 0.05, Fig. 2D and Supplementary Fig. 3D). KDM4A-AS1 knockdown significantly restrained the motility of SK-Hep-1 and MHCC-97H cells (*P* < 0.05, Fig. 2D and Supplementary Fig. 3D). Epithelial–mesenchymal transition (EMT) plays a crucial role in HCC cell migration and invasion. Thus, we detected the epithelial marker (E-cadherin) and the mesenchymal markers (N-cadherin and Vimentin) in HCC cells after altering KDM4A-AS1. Western blotting and immunofluorescence results manifested that KDM4A-AS1 overexpression decreased E-cadherin expression and increased N-cadherin and vimentin expression in Hep3B and Huh7 cells (*P* < 0.05, Fig. 2E and Supplementary Figs. 3E and 4). On the contrary, KDM4A-AS1 knockdown repressed the EMT of SK-Hep-1 and MHCC-97H cells (*P* < 0.05, Fig. 2E and Supplementary Figs. 3E and 4). Thus, these results indicated that KDM4A-AS1 enhanced the proliferation, migration, invasion, and EMT process of HCC cells.

### KDM4A-AS1 promotes HCC growth and metastasis in vivo

**Fig. 3:**
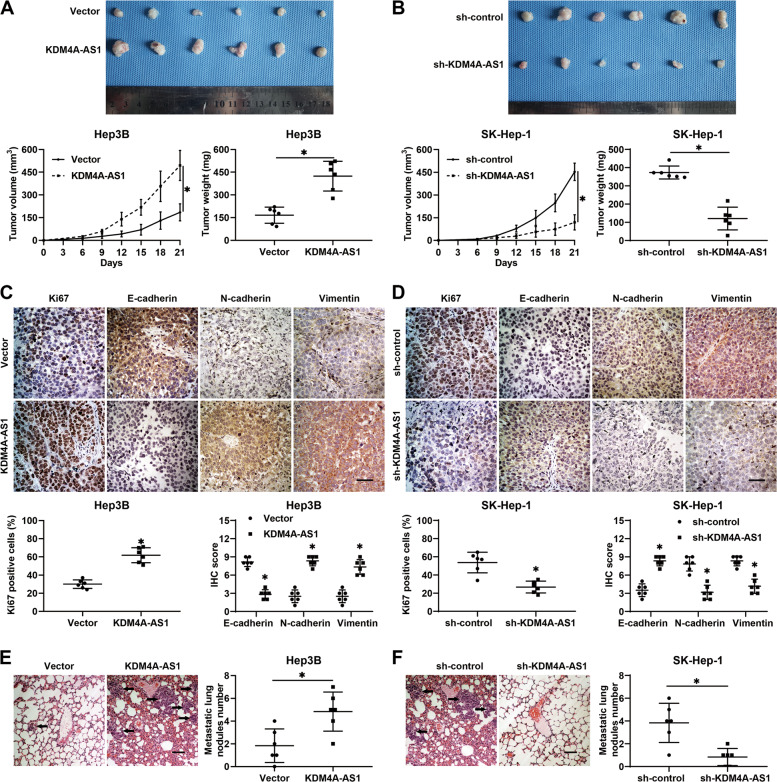
KDM4A-AS1 promotes HCC growth and metastasis in vivo. **A**, **B** The indicated HCC cells were subcutaneously injected into nude mice. Both tumor volume and weight were markedly increased by KDM4A-AS1 overexpression but reduced by KDM4A-AS1 knockdown. **C**, **D** Immunohistochemistry was performed to detect the expression of Ki67, E-cadherin, N-cadherin, and Vimentin in xenograft tumor tissues from different groups. Scale bar: 50 μm. **E**, **F** The indicated HCC cells were injected into the tail vein of nude mice. H&E staining showed that the metastatic nodules in the mouse lung tissues were prominently increased by KDM4A-AS1 overexpression and reduced by KDM4A-AS1 knockdown. Scale bar: 100 μm. All the data are presented as mean ± SD. *P < 0.05.

Next, we further determined the pro-HCC role of KDM4A-AS1 in vivo. Hep3B cells with KDM4A-AS1 overexpression or SK-Hep-1 cells with KDM4A-AS1 knockdown and corresponding control cells were injected subcutaneously into nude mice. The results unraveled that xenograft tumors derived from the KDM4A-AS1 overexpression group were significantly larger than those from the control group (*P* < 0.05, Fig. 3A). In contrast, KDM4A-AS1 knockdown led to HCC growth restriction in mice (*P* < 0.05, Fig. 3B). Immunohistochemistry (IHC) data indicated that KDM4A-AS1 overexpression increased Ki67, N-cadherin, and vimentin staining and decreased E-cadherin staining in xenograft tumor tissues (*P* < 0.05, Fig. 3C). On the contrary, KDM4A-AS1 knockdown decreased Ki67, N-cadherin, and vimentin staining and increased E-cadherin staining (*P* < 0.05, Fig. 3D). In addition, the pulmonary metastatic model was established to examine the function of KDM4A-AS1 in the lung metastasis of HCC. The results manifested that Hep3B cells overexpressing KDM4A-AS1 exhibited more potent metastatic abilities compared to the control cells (*P* < 0.05, Fig. 3E), whereas KDM4A-AS1 knockdown robustly repressed the lung metastasis of SK-Hep-1 cells (*P* < 0.05, Fig. 3F). Hence, these data indicated that KDM4A-AS1 facilitated HCC growth and metastasis in vivo.

### KDM4A-AS1 acts as a molecular sponge for miR-411-5p in HCC cells

**Fig. 4:**
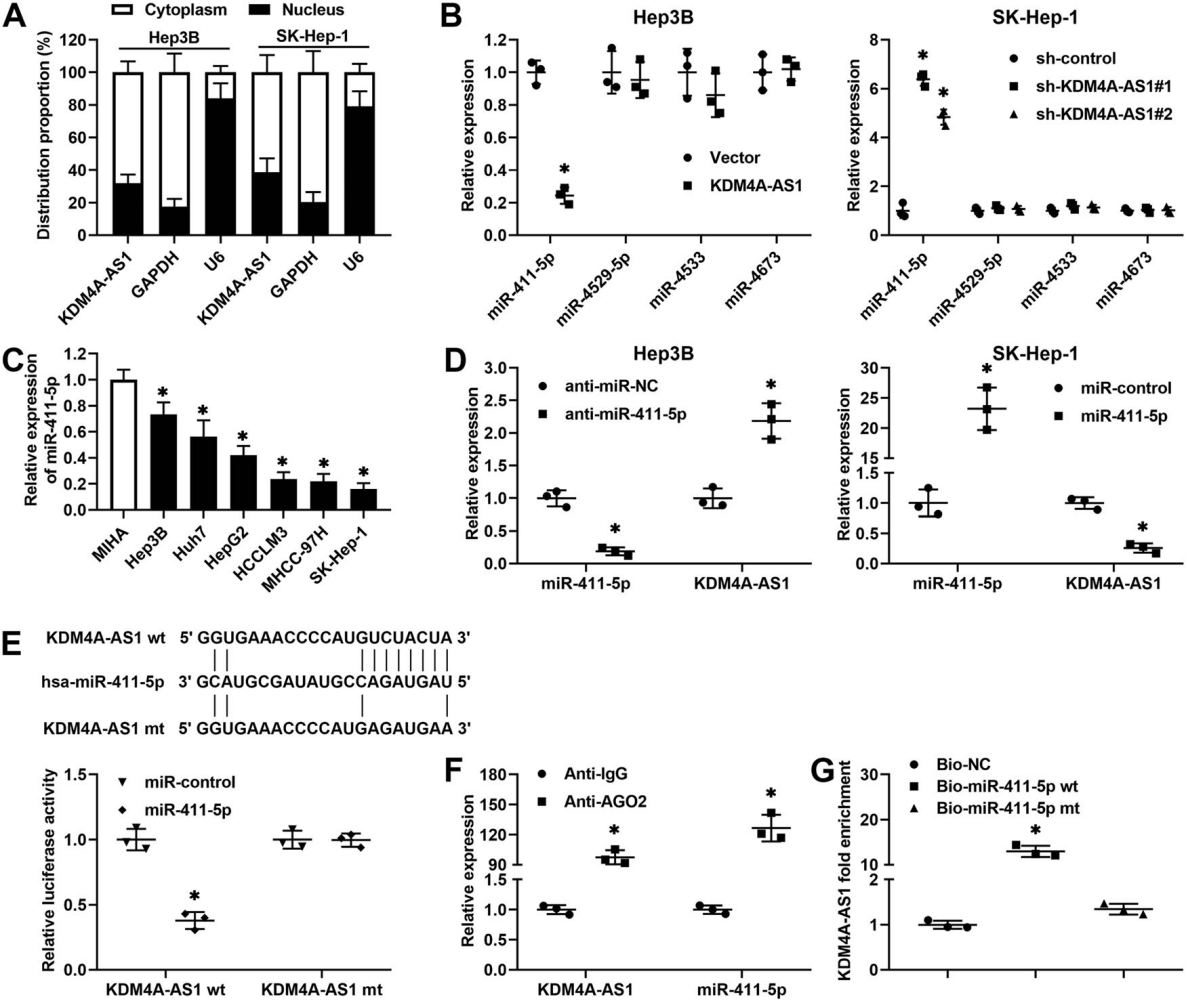
KDM4A-AS1 functions as a molecular sponge of miR-411-5p. **A** A subcellular fractionation assay was conducted to investigate the localization of KDM4A-AS1 in HCC cells. **B** KDM4A-AS1 overexpression reduced and KDM4A-AS1 silencing increased miR-411-5p expression in HCC cells. miR-4529-5p, miR-4533, and miR-4673 levels were not affected by KDM4A-AS1 alteration. **C** The levels of miR-411-5p in HCC cell lines (Hep3B, Huh7, HepG2, HCCLM3, MHCC-97H, SK-Hep-1) and the normal hepatic cell line (MIHA). **D** miR-411-5p depletion increased and miR-411-5p overexpression reduced KDM4A-AS1 expression in HCC cells. **E** The predicted miR-411-5p-binding sites were mutated in KDM4A-AS1. The luciferase activity of HEK293T cells co-transfected with miR-411-5p mimics and wild-type (wt) or mutant (mt) KDM4A-AS1 was measured. **F** Anti-AGO2 RIP was performed in Hep3B cells transiently overexpressing miR-411-5p, and RT-qPCR was carried out to detect miR-411-5p and endogenous KDM4A-AS1 associated with AGO2. **G** KDM4A-AS1 level was determined in samples pulled down by biotinylated wt or mt miR-411-5p. All the data are presented as mean ± SD of at least three independent experiments. *P < 0.05.

The molecular mechanisms involved in the functions of lncRNAs depend on the subcellular localization of themselves. The subcellular fractionation assay and the RNA fluorescent in situ hybridization showed that KDM4A-AS1 was predominantly localized in the cytoplasm rather than in the nucleus of HCC cells (Fig. 4A and Supplementary Fig. 5), inspiring us that KDM4A-AS1 might function as a ceRNA to sequester specific miRNAs and enhance target gene expression. Afterward, miRDB (http://mirdb.org/) and DIANA (LncBase Predicted v.2) bioinformatics tools were used to identify target miRNAs putatively bound to KDM4A-AS1. Four miRNAs were overlapping between these two databases, including miR-411-5p, miR-4529-5p, miR-4533, and miR-4673. To determine which miRNA was the bona fide interaction target of KDM4A-AS1, RT-qPCR was conducted. The results unveiled that only miR-411-5p was negatively regulated by KDM4A-AS1 in HCC cells (*P* < 0.05, Fig. 4B and Supplementary Fig. 6A). RT-qPCR results manifested that the levels of miR-411-5p were downregulated in HCC cell lines compared to MIHA cells (*P* < 0.05, Fig. 4C). In addition, the KDM4A-AS1 expression was negatively regulated by miR-411-5p in HCC cells (*P* < 0.05, Fig. 4D and Supplementary Fig. 6B). The luciferase reporter assay demonstrated that miR-411-5p mimics dramatically reduced the luciferase activity of HEK293T cells transfected with wild-type (wt) KDM4A-AS1, but not with mutant (mt) plasmids (*P* < 0.05, Fig. 4E). Since miRNAs exert biological functions by binding to target mRNAs and inducing RNA degradation and translational repression via forming the RNA-induced silencing complex with AGO2. Hep3B cells transfected with miR-411-5p mimics were subjected to RNA immunoprecipitation (RIP) assay, and the results revealed that both KDM4A-AS1 and miR-411-5p were enriched in anti-AGO2 immunoprecipitated RNA (*P* < 0.05, Fig. 4F). Additionally, KDM4A-AS1 was pulled down by biotin-labeled miR-411-5p, while the interaction between KDM4A-AS1 and miR-411-5p was abolished by the mutation of the miR-411-5p seed region (*P* < 0.05, Fig. 4G). Moreover, miR-411-5p expression was notably downregulated in HCC tissues compared to adjacent non-tumor tissues (*P* < 0.0001, Supplementary Fig. 6C) and negatively correlated with KDM4A-AS1 expression in HCC tissues (*P* = 0.0006, *r* = −0.3533, Supplementary Fig. 6D). Therefore, these results indicated that KDM4A-AS1 functioned as a ceRNA to sponge miR-411-5p.

**Fig. 5:**
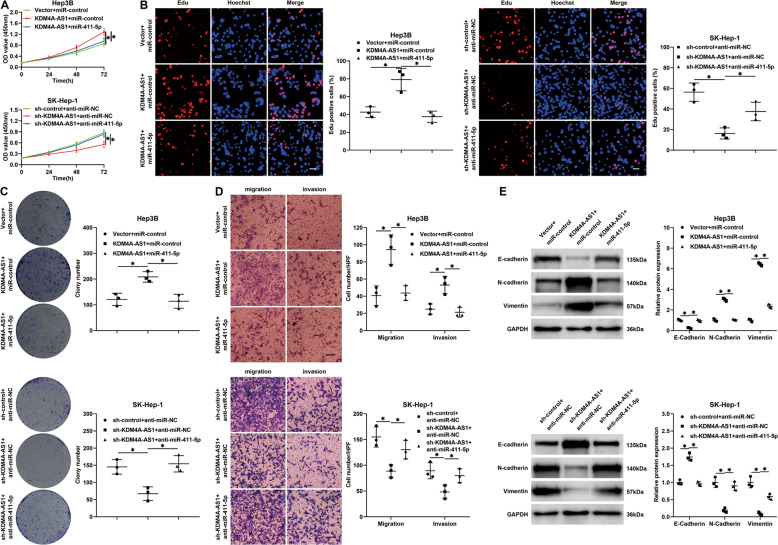
miR-411-5p mediates the oncogenic effects of KDM4A-AS1 on HCC cells. Hep3B cells with KDM4A-AS1 overexpression were transfected with miR-411-5p mimics or control, while SK-Hep-1 cells with KDM4A-AS1 knockdown were transfected with miR-411-5p inhibitors or control. **A** CCK-8, **B** Edu (scale bars = 20 μm), **C** colony formation, **D** transwell (scale bars = 100 μm), and **E** western blotting assays were conducted to examine the proliferation, migration, invasion, and EMT process of HCC cells in different groups. Scale bar: 20 μm for Edu results and 100 μm for transwell results. *P < 0.05.

The previous study has indicated that miR-411-5p functions as a tumor suppressor in HCC [20]. We further examined whether miR-411-5p mediated the oncogenic role of KDM4A-AS1 in HCC. Our data unveiled that miR-411-5p mimics dramatically abolished KDM4A-AS1-enhanced proliferation of Hep3B cells (*P* < 0.05, Fig. 5A–C), while miR-411-5p inhibitors robustly enhanced cell proliferation in SK-Hep-1 cells with KDM4A-AS1 knockdown (*P* < 0.05, Fig. 5A–C). Similarly, the KDM4A-AS1-induced migration, invasion, and EMT of Hep3B cells were abrogated by miR-411-5p (*P* < 0.05, Fig. 5D, E). And miR-411-5p inhibitors restored the KDM4A-AS1 knockdown-repressed motility and EMT of SK-Hep-1 cells (*P* < 0.05, Fig. 5D, E). Collectively, miR-411-5p mediated the role of KDM4A-AS1 in HCC progression.

### Karyopherin α2 (KPNA2) is a novel target of miR-411-5p in HCC

**Fig. 6:**
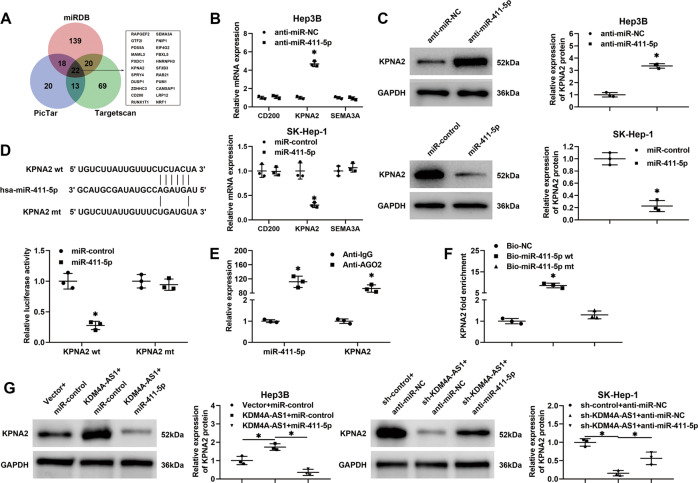
KPNA2 is a direct target of miR-411-5p in HCC cells. **A** Twenty-two potential targets of miR-411-5p were predicted in all three online bioinformatics tools. **B** miR-411-5p inversely regulated KPNA2 mRNA level rather than CD200 and SEMA3A in HCC cells. **C** miR-411-5p silencing upregulated and miR-411-5p overexpression increased KPNA2 protein level in HCC cells. **D** Complementary sequences between miR-411-5p and KPNA2 3’UTR were mutated, and HEK293T cells co-transfected with miR-411-5p mimics and wt or mt KPNA2 3’UTR was detected for luciferase activity. **E** Hep3B cells transfected with miR-411-5p mimics were subjected to anti-AGO2 RIP to detect RNAs associated with AGO2. **F** KPNA2 mRNA level was determined in samples pulled down by biotinylated wt or mt miR-411-5p. **G** Western blotting was performed to detect KPNA2 expression in HCC cells transfected with the indicating vectors. All the data are presented as mean ± SD of at least three independent experiments. *P < 0.05.

To further explore the molecular mechanism underlying the biological function of miR-411-5p in HCC, targetscan, miRDB, and PicTar databases were utilized to predict the potential target genes of miR-411-5p. Twenty-two genes appeared in the predicted results of all three databases (Fig. 6A). Among these genes, only CD200 molecule (CD200) [21], KPNA2 [22–24], and semaphorin 3A [25] are reported to be oncogenes in HCC. RT-qPCR results showed that only KPNA2 was strikingly upregulated by miR-411-5p interference and drastically reduced by miR-411-5p overexpression in HCC cells (*P* < 0.05, Fig. 6B and Supplementary Fig. 7A, B). The regulatory effect of miR-411-5p on the KPNA2 protein level was further confirmed by immunoblotting in HCC cells (*P* < 0.05, Fig. 6C and Supplementary Fig. 7C, D). Luciferase reporter assays manifested that miR-411-5p overexpression significantly repressed the luciferase activity of cells transfected with wt KPNA2 vector, while the mutation of the miR-411-5p-binding site in KPNA2 3’ untranslated region (3’UTR) abolished the effects (*P* < 0.05, Fig. 6D). Furthermore, the RIP assay showed that endogenous KPNA2 was pulled down by an anti-AGO2 antibody accompanied with miR-411-5p in Hep3B cells (*P* < 0.05, Fig. 6E). Moreover, KPNA2 was pulled down by biotinylated wt miR-411-5p but not by mt miR-411-5p (*P* < 0.05, Fig. 6F). In addition, we found that KPNA2 expression was pronouncedly upregulated in HCC tissues compared to adjacent noncancerous tissues (*P* < 0.0001, Supplementary Fig. 8A). KPNA2 was positively associated with KDM4A-AS1 expression (*r* = 0.4960, *P* < 0.0001, Supplementary Fig. 8B) and negatively correlated with miR-411-5p expression (*r* = −0.3867; *P* = 0.0002, Supplementary Fig. 8C) in HCC tissues. Western blotting results confirmed that the KPNA2 level was significantly upregulated in HCC tissues with high KDM4A-AS1 expression than those with low KDM4A-AS1 expression (*P* < 0.05, Supplementary Fig. 8D). Consistently, the expression of KPNA2 was notably upregulated in HCC tissues with low miR-411-5p expression compared to those with high miR-411-5p expression (*P* < 0.05, Supplementary Fig. 8D). Furthermore, the level of KPNA2 was robustly higher in xenograft tumor tissues derived from the KDM4A-AS1 overexpression compared to the control group (*P* < 0.05, Supplementary Fig. 8E). In contrast, KPNA2 expression was drastically lower in xenograft tumor tissues from the KDM4A-AS1 silencing group compared to the control group (*P* < 0.05, Supplementary Fig. 8E). Next, we detected the effect of KDM4A-AS1 on KPNA2 expression in HCC cells. The results showed that KDM4A-AS1 overexpression significantly upregulated KPNA2 expression in Hep3B and Huh7 cells, while miR-411-5p mimics abolished this effect (*P* < 0.05, Fig. 6G and Supplementary Fig. 7E, F). By contrast, KDM4A-AS1 knockdown repressed the KPNA2 level, which was restored by miR-411-5p knockdown in SK-Hep-1 and MHCC-97H cells (*P* < 0.05, Fig. 6G and Supplementary Fig. 7E, F). Altogether, we found that the KDM4A-AS1/miR-411-5p axis modulated KPNA2.

### The KPNA2/AKT pathway mediates the role of KDM4A-AS1 in HCC cells

**Fig. 7:**
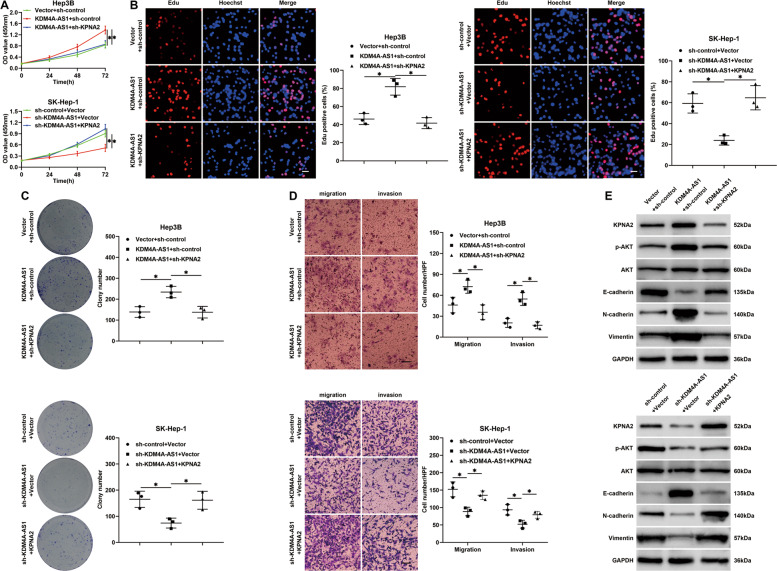
KPNA2 mediates the function of KDM4A-AS1 in HCC cells. Hep3B cells with KDM4A-AS1 overexpression were transfected with KPNA2 shRNA (sh-KPNA2) or nontargeting shRNA (sh-control), while SK-Hep-1 cells with KDM4A-AS1 knockdown were transfected with a vector carrying KPNA2 cDNA or empty vector. **A** CCK-8, **B** Edu, **C** colony formation, and **D** transwell assays were performed to evaluate the proliferation, migration, and invasion of HCC cells in different groups. **E** Western blotting was conducted to measure the levels of KPNA2, p-AKT, AKT, E-cadherin, N-cadherin, and Vimentin in HCC cells. Scale bar: 20 μm for Edu results and 100 μm for transwell results. All the data are presented as mean ± SD of at least three independent experiments. *P < 0.05.

Next, we determined whether KPNA2 mediated the function of KDM4A-AS1 in HCC. Our results revealed that KPNA2 knockdown significantly offset the promoting effects of KDM4A-AS1 on Hep3B cell proliferation (*P* < 0.05, Fig. 7A–C), whereas KPNA2 overexpression rescued the proliferation of SK-Hep-1 cells impeded by KDM4A-AS1 knockdown (*P* < 0.05, Fig. 7A–C). In addition, KPNA2 knockdown diminished KDM4A-AS1-enhanced migration, invasion, and EMT of Hep3B cells (*P* < 0.05, Fig. 7D, E), while KPNA2 overexpression restored the mobility and EMT of SK-Hep-1 cells restrained by KDM4A-AS1 knockdown (*P* < 0.05, Fig. 7D, E). KPNA2 promotes tumor progression by activating the AKT pathway in ovarian and colorectal cancer [26–28]. We further explored whether the AKT pathway was involved in the function of KPNA2 in HCC. The results showed that KDM4A-AS1 overexpression remarkably increased, and KDM4A-AS1 silencing reduced p-AKT level in HCC cells (Fig. 7E). KPNA2 mediated KDM4A-SA1-induced AKT pathway activation in HCC cells (Fig. 7E). Additionally, the AKT inhibitor MK2206 markedly counteracted the promotive effects of KDM4A-AS1 on Hep3B cells (*P* < 0.05, Supplementary Fig. 9). Therefore, these data demonstrated that the KPNA2/AKT pathway participated in KDM4A-AS1-induced malignant behaviors of HCC cells.

### A KDM4A-AS1/KPNA2/HIF-1α signaling loop is found in the hypoxic microenvironment

**Fig. 8:**
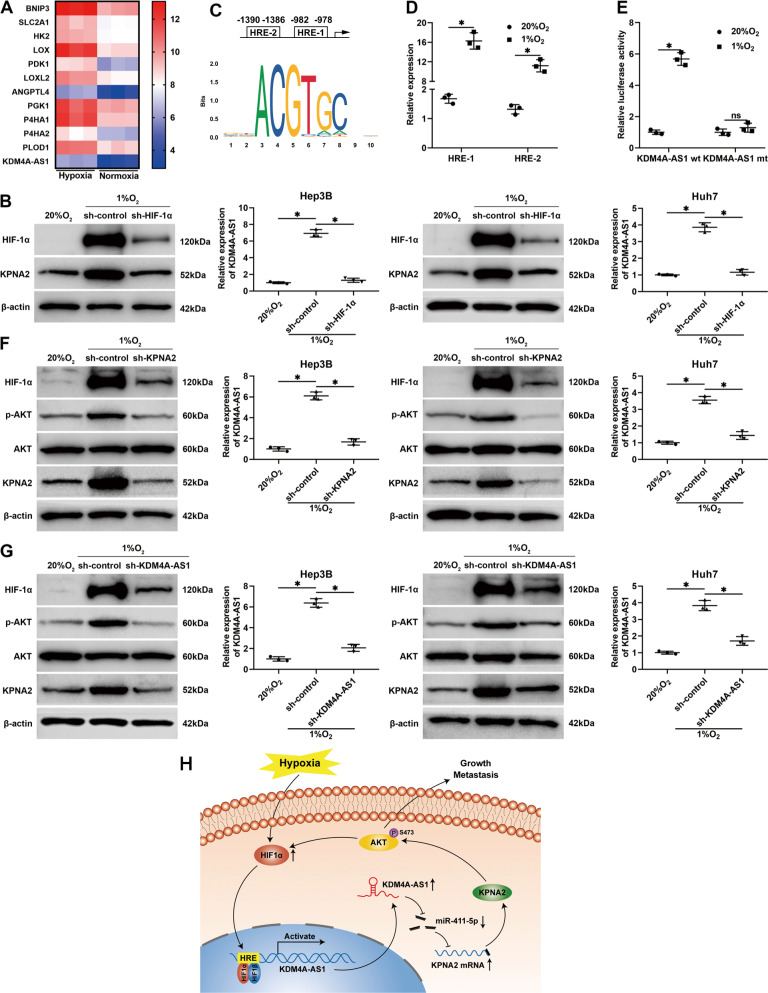
Hypoxia induces a KDM4A-AS1/KPNA2/HIF-1α signaling loop. **A** Heatmap of ten hypoxia-responsive genes and KDM4A-AS1 in Hep3B cells under hypoxia and normoxia. **B** HIF-1α knockdown abolished hypoxia triggered KPNA2 and KDM4A-AS1 upregulation in HCC cells. **C** The HREs in the KDM4A-AS1 promoter region was determined. **D** ChIP assay was conducted to determine the binding between HIF-1α and the HREs of the KDM4A-AS1 promoter. **E** The luciferase reporter plasmid containing wt KDM4A-AS1 promoter or HRE-mutated KDM4A-AS1 promoter was transfected in Hep3B cells. Then, the luciferase activity was measured in Hep3B cells under normoxic or hypoxic conditions. **F** The influence of KPNA2 knockdown on hypoxia-upregulated HIF-1α, KDM4A-AS1, and p-AKT in HCC cells were determined by immunoblotting. **G** The impact of KDM4A-AS1 knockdown on hypoxia-upregulated HIF-1α, KPNA2, and p-AKT in HCC cells was determined by immunoblotting. **H** Schematic of the findings of the present study. All the data are presented as mean ± SD of at least three independent experiments. *P < 0.05.

Emerging evidence has confirmed that hypoxia, a hallmark of the solid tumor, regulates lncRNAs in HCC [12, 14]. Our microarray data (GSE155505) revealed that KDM4A-AS1 expression was upregulated in Hep3B cells under hypoxic conditions (Fig. 8A). Moreover, western blotting results showed that hypoxia upregulated KDM4A-AS1 and KPNA2 expression in a HIF-1α-dependent manner in HCC cells (*P* < 0.05, Fig. 8B). Furthermore, we identified two HREs in the promoter region of KDM4A-AS1 (Fig. 8C). Chromatin immunoprecipitation (ChIP) assay showed that HIF-1α directly bond to the HREs in the KDM4A-AS1 promoter (*P* < 0.05, Fig. 8D). Additionally, luciferase reporter assay revealed that the activity of KDM4A-AS1 promoter was drastically increased by hypoxia, while the mutation of HREs blocked the transactivation of KDM4A-AS1 promoter induced by hypoxia (*P* < 0.05, Fig. 8E). Previous studies confirm that the AKT signaling pathway is involved in modulating HIF-1α in HCC [29, 30]. Western blotting and RT-qPCR results showed that KPNA2 silencing significantly reversed hypoxia-induced HIF-1α, p-AKT, and KDM4A-AS1 levels in HCC cells (*P* < 0.05, Fig. 8F). In addition, KDM4A-AS1 knockdown abolished the upregulation of HIF-1α, p-AKT, and KPNA2 induced by hypoxia (*P* < 0.05, Fig. 8G). Altogether, these data indicated that hypoxia-induced KDM4A-AS1 increased HIF-1α expression by activating the AKT pathway to form a positive signaling loop in HCC.

## Discussion

HCC remains a significant health issue with high mortality and prevalence worldwide. Extensive work has reported that hypoxia, existing ubiquitously in the solid tumor, plays a vital role in the progression of HCC. The hallmark of hypoxia, HIF-1α, which is hydroxylated under the normoxic condition with O_2_ as a substrate and subsequently ubiquitinated for proteasomal degradation, accumulates under hypoxia and functions as a transcription factor to regulate target gene levels, exerting pleiotropic effects on cancer pathobiology, including angiogenesis, metabolism reprogramming, EMT, motility, stemness properties and immune evasion [16]. Emerging evidence indicates that lncRNAs, which play critical roles in tumorigenesis and progression of malignancies, are involved in mediating the biological functions of hypoxia [17, 18]. Our previous studies found that lncRNAs AGAP2-AS1 [10], RUNX1-IT1 [12], and MAPKAPK5-AS1 [14], which were regulated by hypoxia, are involved in the proliferation, motility, EMT, and stemness properties of HCC cells. However, the relationship between hypoxia and various novel lncRNAs remains mysterious, which attracted our attention. This study identified a novel lncRNA KDM4A-AS1, which is drastically overexpressed under hypoxia in Hep3B cells from the microarray data (GSE155505). In addition, the upregulation of KDM4A-AS1 was diminished by the HIF-1α knockdown. Moreover, the ChIP assay and luciferase reporter assay demonstrated that HIF-1α directly bound to and transactivated the KDM4A-AS1 promoter. Thus, we identified a novel hypoxia-induced lncRNA KDM4A-AS1 in HCC.

Next, we found that KDM4A-AS1 was remarkably upregulated in HCC tissues and cell lines. Moreover, high KDM4A-AS1 expression was significantly positively correlated to large tumor size, venous infiltration, and advanced tumor stage. In addition, HCC patients with high KDM4A-AS1 levels suffered a worse prognosis than those with low KDM4A-AS1 levels. These data inspired us that KDM4A-AS1 might serve as an oncogene in HCC and prompted us to conduct more in-depth research on the role of KDM4A-AS1 in HCC. As expected, loss- and gain-of-function experiments revealed that KDM4A-AS1 promoted the proliferation, migration, invasion, and EMT process of HCC cells. Consistently, in vivo experiments validated that KDM4A-AS1 promoted tumor growth and metastasis of HCC. Collectively, our research for the first time unclosed the oncogenic role of KDM4A-AS1 in HCC.

In this study, lncRNA KDM4A-AS1 was identified to be localized in the cytoplasm, which indicated that KDM4A-AS1 might bind to miRNAs to regulate target genes expression. Then, the potential target miRNAs of KDM4A-AS1 were predicted by miRDB and DIANA bioinformatics tools, and miR-411-5p was found to be negatively regulated by KDM4A-AS1. Luciferase reporter assay and RIP assay was conducted further to validate the direct binding between KDM4A-AS1 and miR-411-5p. In addition, we found that miR-411-5p was markedly downregulated in HCC tissues compared to adjacent non-tumor tissues and notably negatively related to KDM4A-AS1 expression. Recent studies have demonstrated the critical role of miR-411-5p in a variety of malignancies. For instance, miR-411-5p accelerates non-small cell lung cancer development by suppressing SPRY4 and TXNIP [31]. In addition, miR-411-5p mediates the promotive function of ECONEXIN in glioma [32]. Moreover, miR-411-5p is negatively regulated by transforming growth factor-β1 and acts as a tumor suppressor via directly downregulating SPRY4 in rhabdomyosarcoma [33]. In HCC, it has been reported that miR-411-5p is sponged by circ_001569 and partially mediated the oncogenic role of circ_001569 [20]. Herein we demonstrated that KDM4A-AS1 accelerated HCC cells proliferation, migration, and invasion via sponging miR-411-5p, which provided us a novel perspective to understand the biological function of miR-411-5p in HCC.

KPNA2 is one of the seven members of the karyopherin α protein family, which plays an essential role in nucleocytoplasmic transport [34]. Previous studies have demonstrated that KPNA2 is dysregulated and plays a vital role in the progression of many malignancies [35]. For instance, KPNA2 promotes epithelial ovarian carcinoma tumor growth via upregulation of c-myc and downregulation of FOXO3a [27]. In addition, KPNA2 contributes to the poor prognosis of breast cancer by aberrant subcellular localization of DNA damage response proteins with subsequent impaired function [36]. Moreover, KPNA2 regulates cellular metabolism through c-myc signaling in glioblastoma [37]. Moreover, KPNA2 promotes tumor development through the nuclear localization of E2F1 and E2F7 in gallbladder cancer [38]. Notably, it has been found that KPNA2 exerts an oncogenic role in HCC via mediating the nuclear import of pleomorphic adenoma gene 1 [22], E2F1 [23], and c-myc [24]. In this research, KPNA2 was identified as a novel target of miR-411-5p in HCC cells. RT-qPCR and western blotting assay showed that miR-411-5p negatively regulated KPNA2 in both RNA and protein levels. Luciferase reporter assay and RIP assay further verify the direct binding between miR-411-5p and KPNA2. Moreover, KPNA2 was overexpressed in HCC tissues compared to corresponding non-tumor tissues and negatively correlated to miR-411-5p while positively correlated to KDM4A-AS1 in HCC tissues. Thus, we identified a KDM4A-AS1/miR-411-5p/KPNA2 axis in HCC. Intriguingly, it has been reported that KPNA2 activates the AKT pathway in various cancers [26–28]. In addition, our previous studies found that the AKT pathway was notably activated under hypoxia [39, 40], which prompted us to investigate whether KPNA2 mediated the effects of hypoxia on AKT activity in HCC. Here, we found that KPNA2 knockdown remarkably reduced the AKT phosphorylation intensified by hypoxia. Additionally, KPNA2/AKT pathway mediates the biological function of KDM4A-AS1 in HCC cells. On the other hand, the AKT pathway is found to be involved in the regulation of HIF-1α [29, 30]. We verified that both KPNA2 knockdown and KDM4A-AS1 knockdown inhibits the HIF-1α upregulation induced by hypoxia. Therefore, we revealed a hypoxia-triggered KDM4A-AS1/KPNA2/HIF-1α signaling loop that promoted HCC growth and metastasis.

To conclude, we identified a novel HIF-1α-activated lncRNA KDM4A-AS1 in HCC. Meanwhile, KDM4A-AS1 was aberrantly overexpressed in HCC, and the high KMD4A-AS1 level was relevant to unfavorable clinical features and poor prognosis. Additionally, KDM4A-AS1 contributed to the growth and metastasis of HCC via the miR-411-5p/KPNA2/AKT axis. KPNA2 increased HIF-1α level by activating the AKT pathway to form a KDM4A-AS1/KPNA2/HIF-1α positive feedback loop under hypoxia in HCC (Fig. 8H). Altogether, KDM4A-AS1 acted as an oncogene in HCC occurrence and progression and might be a valuable prognostic marker and potential therapeutic target for HCC.

## Materials and methods

### Patients and clinical specimens

Ninety pairs of HCC and adjacent non-tumor tissues were obtained from patients who underwent hepatectomy at the First Affiliated Hospital of Xi’an Jiaotong University (Xi’an, China). The patients receiving any preoperative treatment were excluded. We obtained written informed consent from all participants. Tumor tissues, as well as the adjacent non-tumor tissues, were snap-frozen in liquid nitrogen and stored at −80 °C. We delineated the clinicopathological and demographic information of the patients in Supplementary Table 1. All procedures involving human participants were in accordance with the ethical standards of the Research Ethics Committee of The First Affiliated Hospital of Xi’an Jiaotong University and with the Declaration of Helsinki as revised in 2013. Written informed consent to participate in the study was obtained from patients with HCC prior to sample collection.

### Cell culture

The HCC cell lines (Hep3B, Huh7, HepG2, MHCC-97H, HCCLM3, SK-Hep-1), the normal hepatic cell line (MIHA), and HEK293T cells were maintained in our lab [9, 14]. All cells are authenticated by STR profiling and tested for mycoplasma contamination every 3–6 months. All cells were cultured in Dulbecco’s modified Eagle’s medium (Gibco; Thermo Fisher Scientific, Inc., Waltham, MA, USA) supplemented with 10% fetal bovine serum (Gibco; Thermo Fisher Scientific, Inc.), and 1% penicillin–streptomycin (Gibco; Thermo Fisher Scientific, Inc.) in a humidified 5% CO_2_ incubator at 37 °C.

### Plasmids and transfection

KDM4A-AS1 shRNAs (sh-KDM4A-AS1#1, sh-KDM4A-AS1#2 and sh-KDM4A-AS1#3), KPNA2 shRNA, and scrambled shRNA (sh-control) were designed and synthesized by Shanghai GenePharma Co., Ltd. (Shanghai, China). pcDNA3.1-KDM4A-AS1 (KDM4A-AS1) and pcDNA3.1-control (Vector) were obtained from Invitrogen Corporation (Carlsbad, CA, USA). miR-411-5p mimics (HmiR-SN0486), miR-411-5p inhibitors (HmiR-AN0486-SN-10), miRNA mimics negative control (CmiR-SN0001-SN), and miRNA inhibitor negative control (CmiR-AN0001-SN) were purchased from GeneCopoeia, Inc. (Guangzhou, China). The KPNA2 Human cDNA ORF Clone (KPNA2) was purchased from OriGene (OriGene Technologies, Inc., Beijing, China). HIF-1α shRNA and scrambled shRNA (sh-control) was purchased from GeneCopoeia, Inc. Cell transfection was performed as previously described [9].

### RNA extraction and RT-qPCR

According to the manufacturer’s instructions, RNA was extracted from tissues and cells using TRIzol reagent (Invitrogen). cDNA was synthesized using a cDNA Synthesis kit (Thermo Fisher Scientific, Inc.). All miRNAs were reverse transcribed into cDNA with a TaqMan MicroRNA Reverse Transcription Kit (Applied Biosystems, Foster City, CA, USA). RT-qPCR was performed with SYBR® Green Premix PCR Master Mix (Roche Diagnostics, Indianapolis, IN, USA). The sequences of the primers used are listed in Supplementary Table 2.

### Cell proliferation assays

CCK-8 assay, plate clone formation assay, and Edu assay were conducted to determine cells’ proliferation ability. These assays were conducted as reported previously [9, 41].

### Transwell assays

Transwell migration and invasion assays were performed to detect cell migration and invasion ability according to the protocols described in our previous studies [41].

### Western blotting

The assay was conducted according to the protocols as described previously [41]. The antibodies used in this study are listed in Supplementary Table 3.

### Immunofluorescence

HCC cells were fixed with 4% paraformaldehyde and permeabilized with 0.2% Triton X-100. Then, the cells were incubated with the E-cadherin and vimentin primary antibodies overnight at 4 °C and corresponding secondary antibodies with an Alexa Fluor-conjugated IgG (Yeasen Biotechnology Co., Ltd., Shanghai, China). Fluorescence images were captured using a Zeiss fluorescence photomicroscope (Carl Zeiss AG).

### In vivo experiments

The Male BALB/c nude mice (4 weeks old) were housed under pathogen-free conditions in the Centre of Laboratory Animals at The Medical College of Xi’an Jiaotong University. Animal experiments were performed according to the protocols approved by the Ethics Review Committee of Xi’an Jiaotong University. Mice were randomly grouped (*n* = 6 mice per group, the sample size is based on experience from previous studies using the same animals) by random number method with no blinding. A mouse model was established via injecting HCC cells (3 × 10^6^) subcutaneously into the flank of the mice, and we determined the tumor volume for each mouse every three days using 0.5 × length × width × width. The mice were sacrificed after 3 weeks and the tumor specimens were harvested for further experiments. The pulmonary metastatic model was established by injecting HCC cells (1 × 10^6^) into the tail vein. After 6 weeks, the lung tissues were collected and subjected to hematoxylin and eosin staining, followed by examination microscopically.

### IHC staining

Formalin-fixed and paraffin-embedded tissues were cut into sections, dewaxed in xylene, rehydrated with ethanol of decreasing concentration, and subsequently boiled in retrieval solution to expose antigens. After that, the slides were incubated with the primary antibodies against Ki67, E-cadherin, N-cadherin, and Vimentin overnight at 4 °C and corresponding secondary antibodies coupling with horseradish peroxidase for 10 min at room temperature. Next, diaminobenzidine was reacted under horseradish peroxidase catalyzation, leading to brown pigment at the site. Then, the slides were counterstained with hematoxylin and inspected under a microscope. The results of Ki67 staining were analyzed by positive staining cell percentage. The IHC scores, defined as staining intensity score (none scored 0; weak scored 1; moderate scored 2; strong scored 3) × percentage score (0 for <5%; 1 for 5–25%; 2 for 25–50%; 3 for >50%), were used to assess the results of E-cadherin, N-cadherin, and Vimentin staining. The antibodies used in this study are listed in Supplementary Table 3.

### Subcellular fractionation

The PARIS Kit (Life Technologies, Inc., Carlsbad, CA, USA) was applied for nuclear and cytoplasmic isolation. Briefly, 1 × 10^7^ cells were collected and washed once in phosphate-buffered saline, and then resuspended and incubated for 10 min in 300 μl Cell Fractionation Buffer. After centrifugation, the supernatant containing the cytoplasmic fraction was aspirated and collected while the pellet containing the nuclear fraction was resuspended in 300 μl Cell Disruption Buffer. The buffer containing cytoplasmic/nuclear fraction was subjected to RNA extraction according to the manufacturer’s instructions, respectively.

### RNA fluorescent in situ hybridization (FISH)

Subcellular localization of KDM4A-AS1 was determined by the FISH kit (RiboBio, Guangzhou, China) according to the manufacturer’s procedure. In brief, Hep3B cells were plated on glass coverslips into 24-well plates. After being fixed by 4% paraformaldehyde and permeabilized by 0.5% Triton X-100, the cells were incubated with prehybridization solution and hybridized with hybridization solution and then incubated with Cy3-labeled KDM4A-AS1 oligonucleotide probe overnight. Cell nuclei were stained with 4,6-diamidino-2-phenylindole. Images were captured under a Zeiss fluorescence photomicroscope (Carl Zeiss AG).

### Luciferase reporter assay

The wt or mt sequences of KDM4A-AS1 or KPNA2 3’UTR were synthesized and inserted into pGL3 vectors (Promega Corporation, Madison, WI, USA), respectively. Subsequently, the vectors were transfected into HEK293T cells accompanied by miRNA mimics or control. pGL3-basic vector carrying wt KDM4A-AS1 promoter or HRE-mutated KDM4A-AS1 promoter was named KDM4A-AS1 wt or mt. After transfection with these vectors, HEK293T cells were under hypoxia for 48 h. We utilized the Dual-Luciferase Reporter Assay System (Promega Corporation) to detect the relative luciferase activities and used Renilla luciferase activity for normalization.

### RIP assay

RIP assay was conducted with the EZ-Magna RIP Kit (Millipore, Billerica, MA, USA). Briefly, cells lysed in complete RIP lysis buffer were incubated with magnetic beads conjugated to antibodies against Ago2 (Millipore), followed by proteinase K incubation to degrade proteins. Then, the immunoprecipitated RNA was analyzed with an RT-qPCR assay.

### Biotin pull-down assay

The wt and mt miR-411-5p were labeled with biotin to obtain Bio-miR-411-5p wt and Bio-miR-411-5p mt (Guangzhou RiboBio Co., Ltd), which were transfected into HCC cells subsequently. After 48 h, cells were lysed and incubated with Dynabeads M-280 Streptavidin (Invitrogen) for 3 h at 4 °C. Afterward, the beads were washed with lysis buffer three times and high salt buffer for once. The bound RNAs were subjected to RT-qPCR assay.

### Chromatin immunoprecipitation

ChIP assays were conducted following the manufacturing procedure using SimpleChIP® Enzymatic Chromatin IP Kit (Cell Signaling Technology, Beverly, MA, USA). After incubation in normoxia and hypoxia for 16 h, cells were cross-linked in formaldehyde. Then, chromatin was sonicated, and the lysates were incubated with antibodies against HIF-1α or IgG. RT-qPCR analyzed the immunoprecipitated chromatins. The used primers are listed in Supplementary Table 2.

### Statistical analysis

Data are presented as mean ± SD. We used theGraphPad Prism software version 8.0 (GraphPad Software, Inc., San Diego, CA, USA) for statistical analysis. Data were compared using a two-tailed Student’s *t* test and analysis of variance test. The OS between the two groups was analyzed using Kaplan–Meier curves and log-rank analysis. The association between KDM4A-AS1 expression and clinicopathological features was analyzed using Chi-squared test and Fisher’s exact test. We performed Pearson correlation analysis to determine the correlation between KDM4A-AS1, miR-411-5p, and KPNA2 expression. *P* < 0.05 was considered to indicate a statistically significant difference.

## Supplementary information

Supplementary Table 1

Supplementary Table 2

Supplementary Table 3

Supplementary Figure Legends

Supplementary Figure 1

Supplementary Figure 2

Supplementary Figure 3

Supplementary Figure 4

Supplementary Figure 5

Supplementary Figure 6

Supplementary Figure 7

Supplementary Figure 8

Supplementary Figure 9

checklist

## Data Availability

The datasets used and analyzed during the current study are available from the corresponding author on reasonable request.
