# Supplementary material for: HIF-1α-activated long non-coding RNA KDM4A-AS1 promotes hepatocellular carcinoma progression via the miR-411-5p/KPNA2/AKT pathway
Source: Cell Death Dis. 2021 Dec 13;12(12):1152. doi: 10.1038/s41419-021-04449-2 (PMC8668937; doi:10.1038/s41419-021-04449-2)
Supplement: Supplementary file 1 — Supplementary Table 1 [file 41419_2021_4449_MOESM1_ESM.docx]

**Supplementary Table** **1** Clinical correlation analysis of KDM4A-AS1 expression in human hepatocellular carcinoma

| **Clinical parameters** | **Expression level** | | ***P-*value** |
| --- | --- | --- | --- |
|  | **KDM4A-AS1^high^(n=45)** | **KDM4A-AS1^low^(n=45)** |  |
| Age (years) |  |  | 0.490 |
| < 50 | 12 | 15 |  |
| ≥ 50 | 33 | 30 |  |
| Gender |  |  | 0.353 |
| Male | 37 | 41 |  |
| Female | 8 | 4 |  |
| Tumor size (cm) |  |  | 0.003 |
| < 5 | 16 | 30 |  |
| ≥ 5 | 29 | 15 |  |
| Tumor number |  |  | 0.378 |
| solitary | 31 | 27 |  |
| multiple | 14 | 18 |  |
| TNM stage |  |  | 0.009 |
| Ⅰ+Ⅱ | 30 | 41 |  |
| Ⅲ+Ⅳ | 15 | 4 |  |
| Venous infiltration |  |  | 0.011 |
| Present | 26 | 14 |  |
| Absent | 19 | 31 |  |
| AFP (ng/ml) |  |  | 0.809 |
| <20 | 11 | 12 |  |
| ≥20 | 34 | 33 |  |
| HBV infection |  |  | 0.128 |
| Yes | 38 | 32 |  |
| No | 7 | 13 |  |
