# Supplementary material for: HIF-1α-activated long non-coding RNA KDM4A-AS1 promotes hepatocellular carcinoma progression via the miR-411-5p/KPNA2/AKT pathway
Source: Cell Death Dis. 2021 Dec 13;12(12):1152. doi: 10.1038/s41419-021-04449-2 (PMC8668937; doi:10.1038/s41419-021-04449-2)
Supplement: Supplementary file 2 — Supplementary Table 2 [file 41419_2021_4449_MOESM2_ESM.docx]

**Supplementary Table 2** List of primers used in the study

| **Gene** | **Primer sequences (5’-3’)** |
| --- | --- |
| KDM4A-AS1-F | AGGGTGAAAGGAACGTCCAC |
| KDM4A-AS1-R | TGAAGTACTTTGCCAGGTCCC |
| miR-411-5p-F | CCGGAACCCCCTCCTTACTC |
| miR-411-5p-R | AATGGGATGTGTCCGAAGGA |
| KPNA2-F | ATTGCAGGTGATGGCTCAGT |
| KPNA2-R | CTGCTCAACAGCATCTATCG |
| β-Actin-F | ACTCGTCATACTCCTGCT |
| β-Actin-R | GAAACTACCTTCAACTCC |
| U6-F | CTCGCTTCGCRCAGCACA |
| U6-R | AACGCTTCACGAATTTGCGT |
| KDM4A-AS1 HRE1-F | CAAGAGGCGTTCCATTGATT |
| KDM4A-AS1 HRE1-R | AAAGGAAGCTGGCACAGAAA |
| KDM4A-AS1 HRE2-F | CTTCACTGGTCCTTCCCTCA |
| KDM4A-AS1 HRE2-R | GTTGTGTCCTCGGTGCTTTT |
