# Supplementary material for: HIF-1α-activated long non-coding RNA KDM4A-AS1 promotes hepatocellular carcinoma progression via the miR-411-5p/KPNA2/AKT pathway
Source: Cell Death Dis. 2021 Dec 13;12(12):1152. doi: 10.1038/s41419-021-04449-2 (PMC8668937; doi:10.1038/s41419-021-04449-2)
Supplement: Supplementary file 3 — Supplementary Table 3 [file 41419_2021_4449_MOESM3_ESM.docx]

**Supplementary Table 3** List of antibodies used in the study

| **Antibody** | **Source** | **Catalog** |
| --- | --- | --- |
| E-Cadherin | Cell Signaling Technology | #3195 |
| N-Cadherin | Cell Signaling Technology | #13116 |
| Vimentin | Cell Signaling Technology | #5741 |
| KPNA2 | Santa Cruz Biotechnology | sc-55538 |
| Akt | Cell Signaling Technology | #4691 |
| Phospho-Akt (Ser473) | Cell Signaling Technology | #4060 |
| Ki67 | Abcam | ab92742 |
| HIF-1α | Cayman Chemical Company | 10006421 |
| β-Actin | proteintech | 66009-1-Ig |
| GAPDH | Abcam | ab181602 |
