# Supplementary material for: HIF-1α-activated long non-coding RNA KDM4A-AS1 promotes hepatocellular carcinoma progression via the miR-411-5p/KPNA2/AKT pathway
Source: Cell Death Dis. 2021 Dec 13;12(12):1152. doi: 10.1038/s41419-021-04449-2 (PMC8668937; doi:10.1038/s41419-021-04449-2)
Supplement: Supplementary file 4 — Supplementary Figure Legends [file 41419_2021_4449_MOESM4_ESM.docx]

**Supplementary figure legends**

**Supplementary Figure 1 The levels of KDM4A-AS1 in HCC cell lines (Hep3B, Huh7, HepG2, HCCLM3, MHCC-97H, SK-Hep-1) and the normal hepatic cell line (MIHA).** *P<0.05

**Supplementary Figure 2 The transfection efficiency of indicating vectors in HCC cells.** (A and B) RT-qPCR was performed to determine the KDM4A-AS1 expression in Hep3B and Huh7 cells transfected with a vector containing KDM4A-AS1 or empty vector. (C and D) RT-qPCR was conducted to detect the KDM4A-AS1 expression in SK-Hep-1 and MHCC-97H cells transfected with KDM4A-AS1 shRNA (sh-KDM4A-AS1#1, sh-KDM4A-AS1#2, sh-KDM4A-AS1#3) or scrambled shRNA (sh-control). *P<0.05

**Supplementary Figure 3 KDM4A-AS1 promotes HCC cell proliferation, migration, and invasion.** (A) CCK-8 assay was performed to determine the effects of KDM4A-AS1 overexpression or knockdown on HCC cell viability. (B) Colony formation assay was utilized to evaluate the function of KDM4A-AS1 in HCC cell proliferation. (C) Edu assay was carried out to explore the proliferation of HCC cells affected by KDM4A-AS1 overexpression or knockdown. Scale bar: 20μm. (D) The effects of KDM4A-AS1 on the migration and invasion abilities of HCC cells were detected by transwell assay. Scale bar: 100μm. (E) E-cadherin, N-cadherin, and Vimentin levels were detected to identify the EMT process of HCC cells affected by KDM4A-AS1 overexpression or knockdown. *P<0.05

**Supplementary Figure 4 KDM4A-AS1 enhanced EMT progression of HCC cell.** Immunofluorescence assay was performed to compare the expression patterns of E-cadherin and Vimentin. The nuclei were stained with DAPI. Scale bar: 20μm

**Supplementary Figure 5 The subcellular location of KDM4A-AS1 in KDM4A-AS1-overexpressing Hep3B cells was determined by FISH assay.** Scale bar: 20μm

**Supplementary Figure 6 miR-411-5p inversely regulates KDM4A-AS1 expression in HCC.** (A) KDM4A-AS1 overexpression reduced miR-411-5p expression in Huh7 cells, and KDM4A-AS1 silencing increased miR-411-5p level in MHCC-97H cells. (B) miR-411-5p depletion increased, and miR-411-5p overexpression reduced KDM4A-AS1 expression in HCC cells. (C) RT-qPCR was performed to detect miR-411-5p expression in 90 pairs of HCC and corresponding adjacent non-tumor tissues. (D) Pearson’s correlation analysis was utilized to determine the correlation between KDM4A-AS1 and miR-411-5p expression in HCC tissues. *P<0.05

**Supplementary Figure 7 KPNA2 is a target of miR-411-5p in HCC.** (A and B) RT-qPCR was carried out to measure KPNA2 mRNA expression in HCC cells transfected with miR-411-5p mimics or inhibitors. (C and D) Western blotting was conducted to explore the KPNA2 protein level in HCC cells transfected with miR-411-5p mimics or inhibitors. (E and F) Western blotting was performed to detect KPNA2 expression in Huh7 and MHCC-97H cells co-transfected vectors. *P<0.05

**Supplementary Figure 8 The correlations among KPNA2, miR-411-5p, and KDM4A-AS1 expression in HCC.** (A) The different expression of KPNA2 mRNA between HCC and adjacent non-tumor tissues. (B) The positive correlation between KPNA2 mRNA and KDM4A-AS1 expression was determined in HCC tissues. (C) A negative association between KPNA2 mRNA and miR-411-5p was confirmed in HCC tissues. (D) The KPNA2 protein level in HCC tissues with high KDM4A-AS1 or low miR-411-5p was higher than HCC samples with low KDM4A-AS1 or high miR-411-5p levels. (E) KPNA2 protein expression in xenograft tumor tissues was upregulated in the KDM4A-AS1 overexpression group and reduced in the KDM4A-AS1 group compared to the control group. *P<0.05

**Supplementary Figure 9 The function of KDM4A-AS1 is mediated by the AKT pathway.** Hep3B cells with KDM4A-AS1 overexpression were treated with AKT inhibitor MK2206. (A) CCK8, (B) Edu, (C) colony formation, and (D) transwell assays were employed to explore the proliferation, migration, and invasion of Hep3B cells in different groups. (E) Western blotting was conducted to measure p-AKT, AKT, E-cadherin, N-cadherin, and Vimentin in HCC cells. *P<0.05
