# Supplementary figures and images for: HIF-1α-activated long non-coding RNA KDM4A-AS1 promotes hepatocellular carcinoma progression via the miR-411-5p/KPNA2/AKT pathway
Source: Cell Death Dis. 2021 Dec 13;12(12):1152. doi: 10.1038/s41419-021-04449-2 (PMC8668937; doi:10.1038/s41419-021-04449-2)

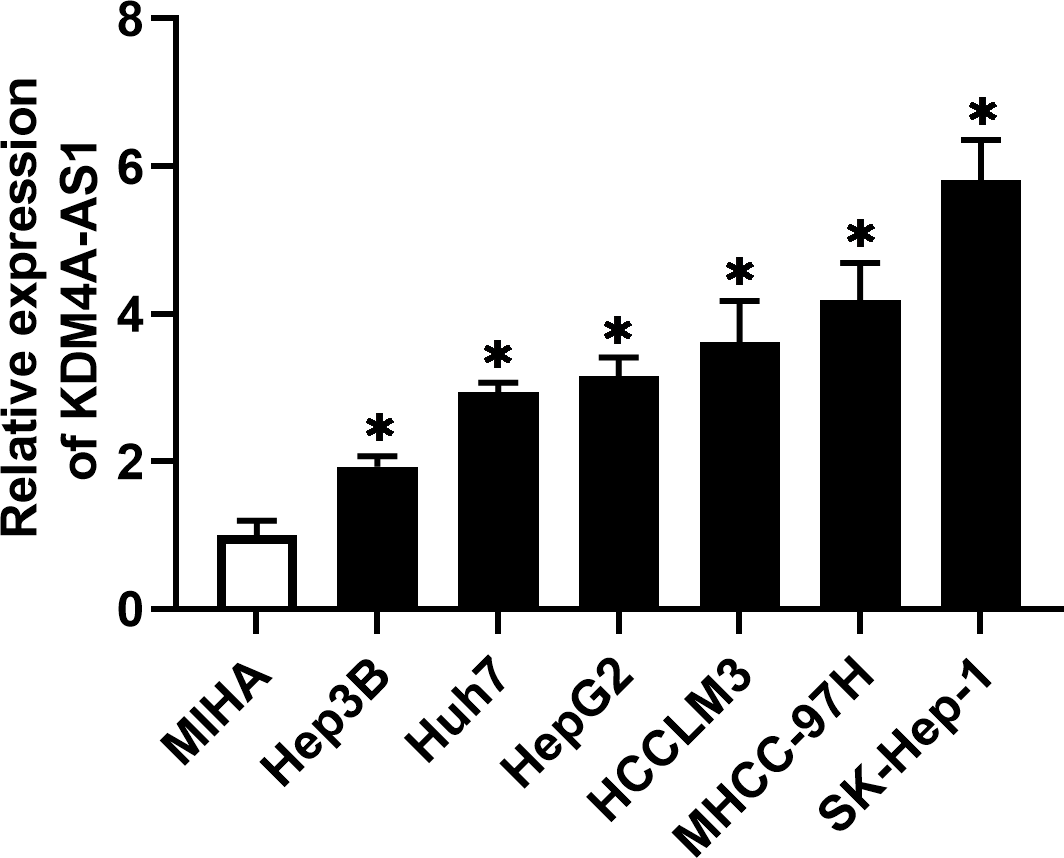

Supplement: Supplementary file 5 — Supplementary Figure 1 [file 41419_2021_4449_MOESM5_ESM.tif]

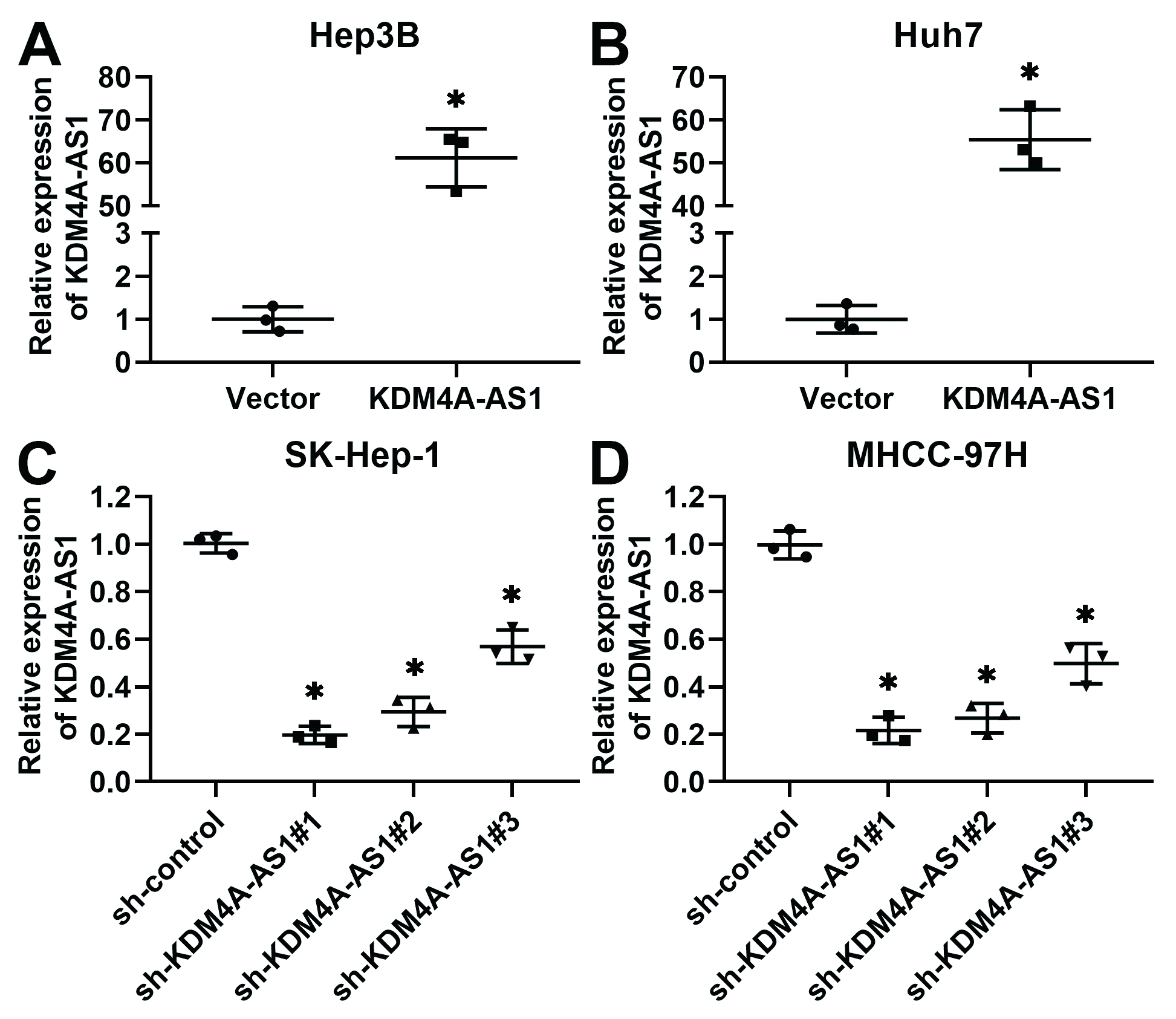

Supplement: Supplementary file 6 — Supplementary Figure 2 [file 41419_2021_4449_MOESM6_ESM.tif]

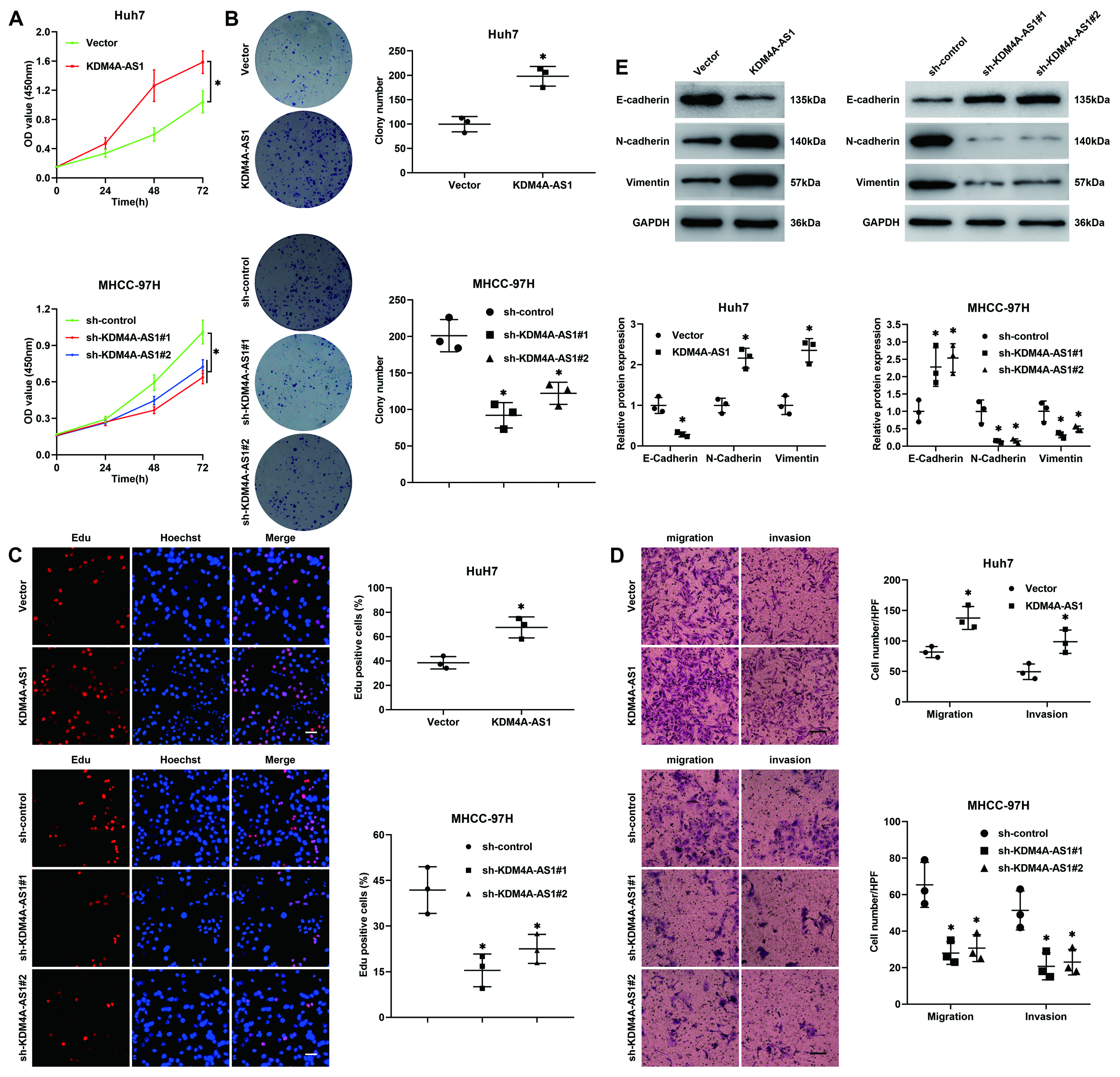

Supplement: Supplementary file 7 — Supplementary Figure 3 [file 41419_2021_4449_MOESM7_ESM.tif]

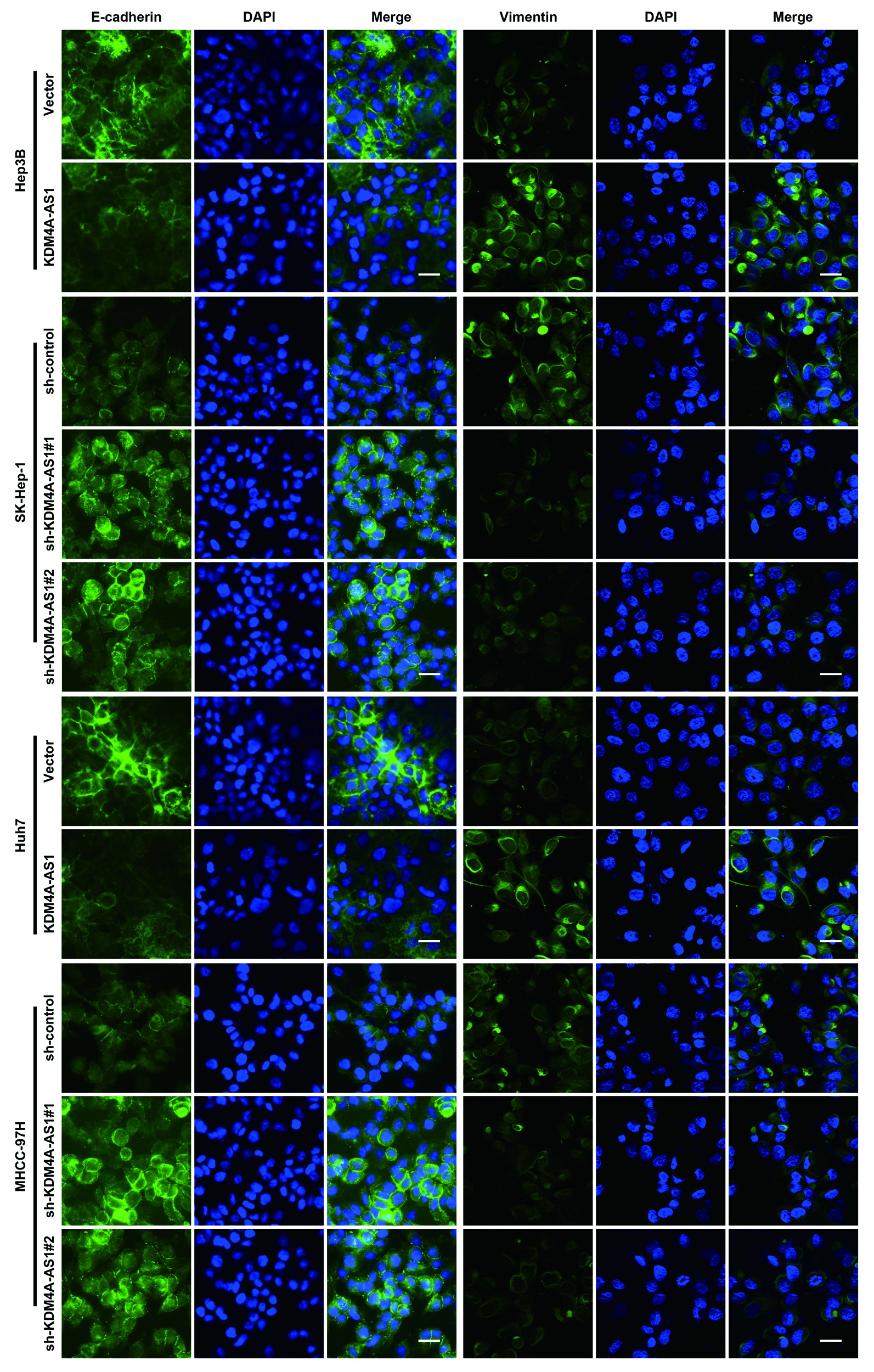

Supplement: Supplementary file 8 — Supplementary Figure 4 [file 41419_2021_4449_MOESM8_ESM.tif]

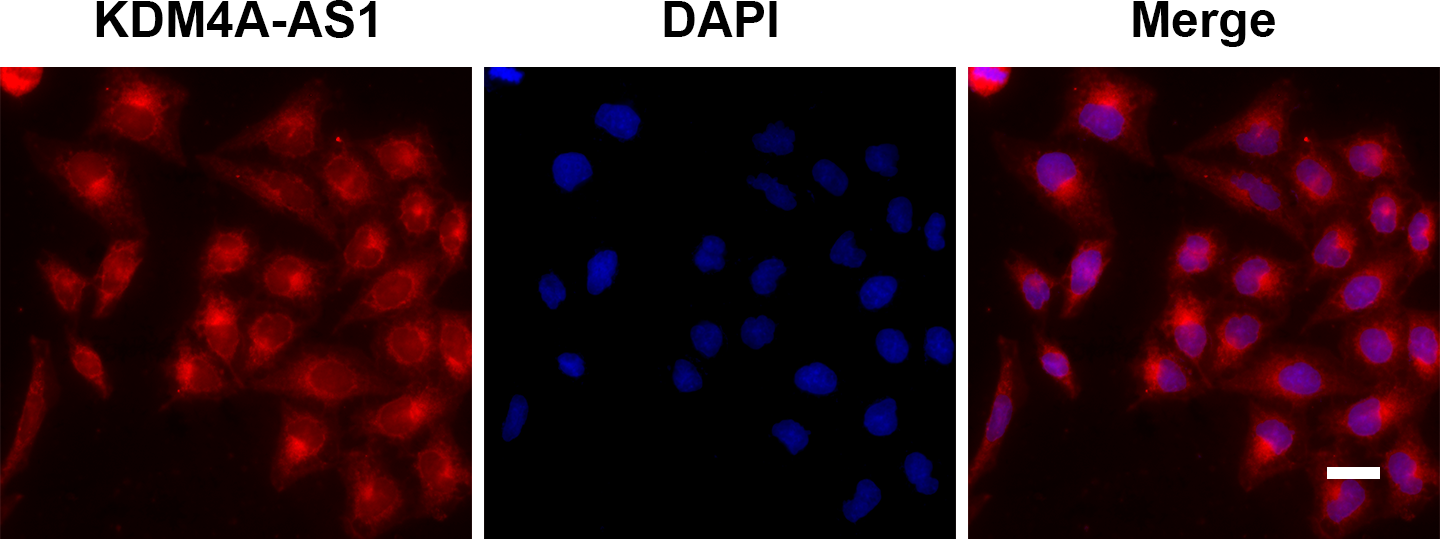

Supplement: Supplementary file 9 — Supplementary Figure 5 [file 41419_2021_4449_MOESM9_ESM.tif]

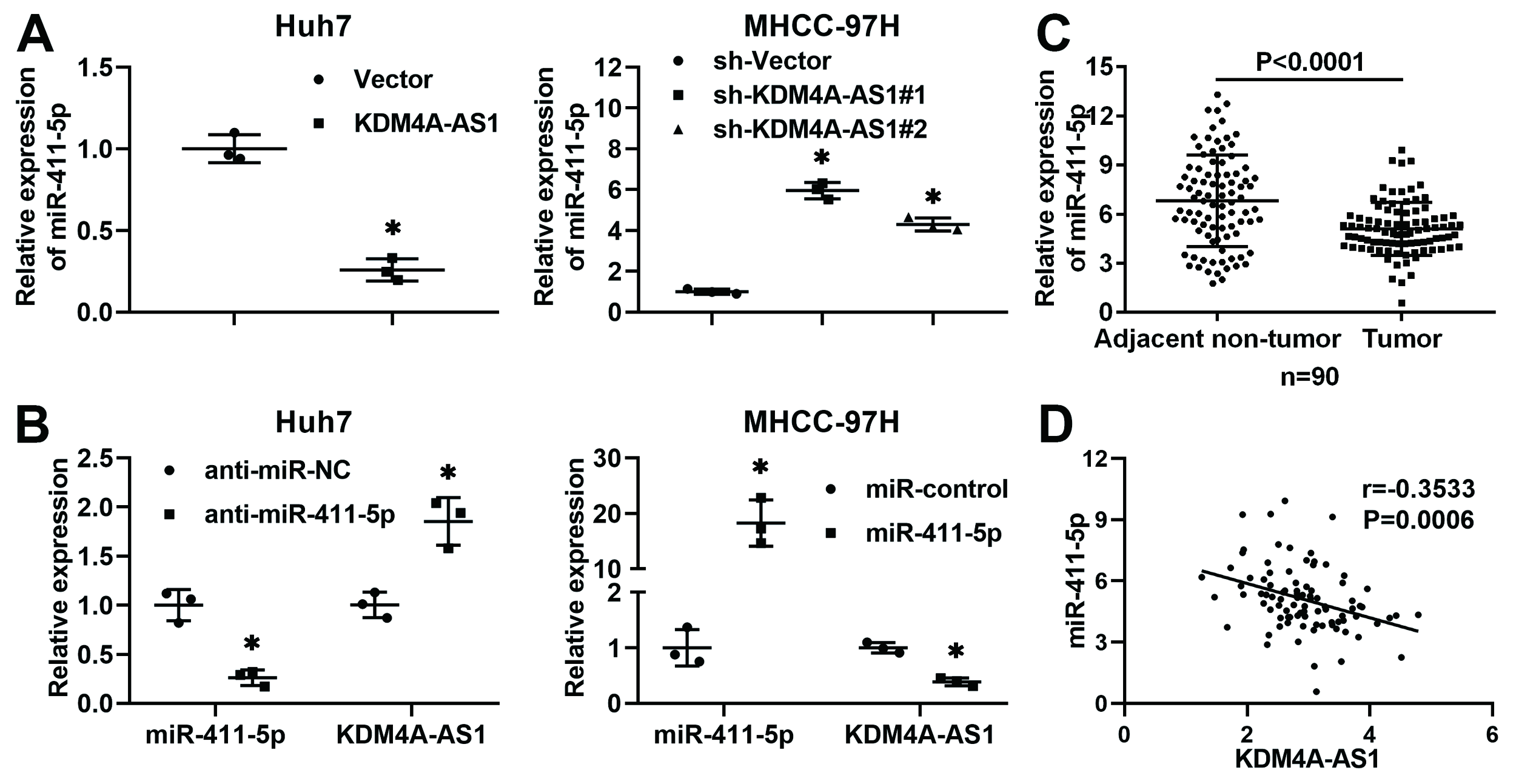

Supplement: Supplementary file 10 — Supplementary Figure 6 [file 41419_2021_4449_MOESM10_ESM.tif]

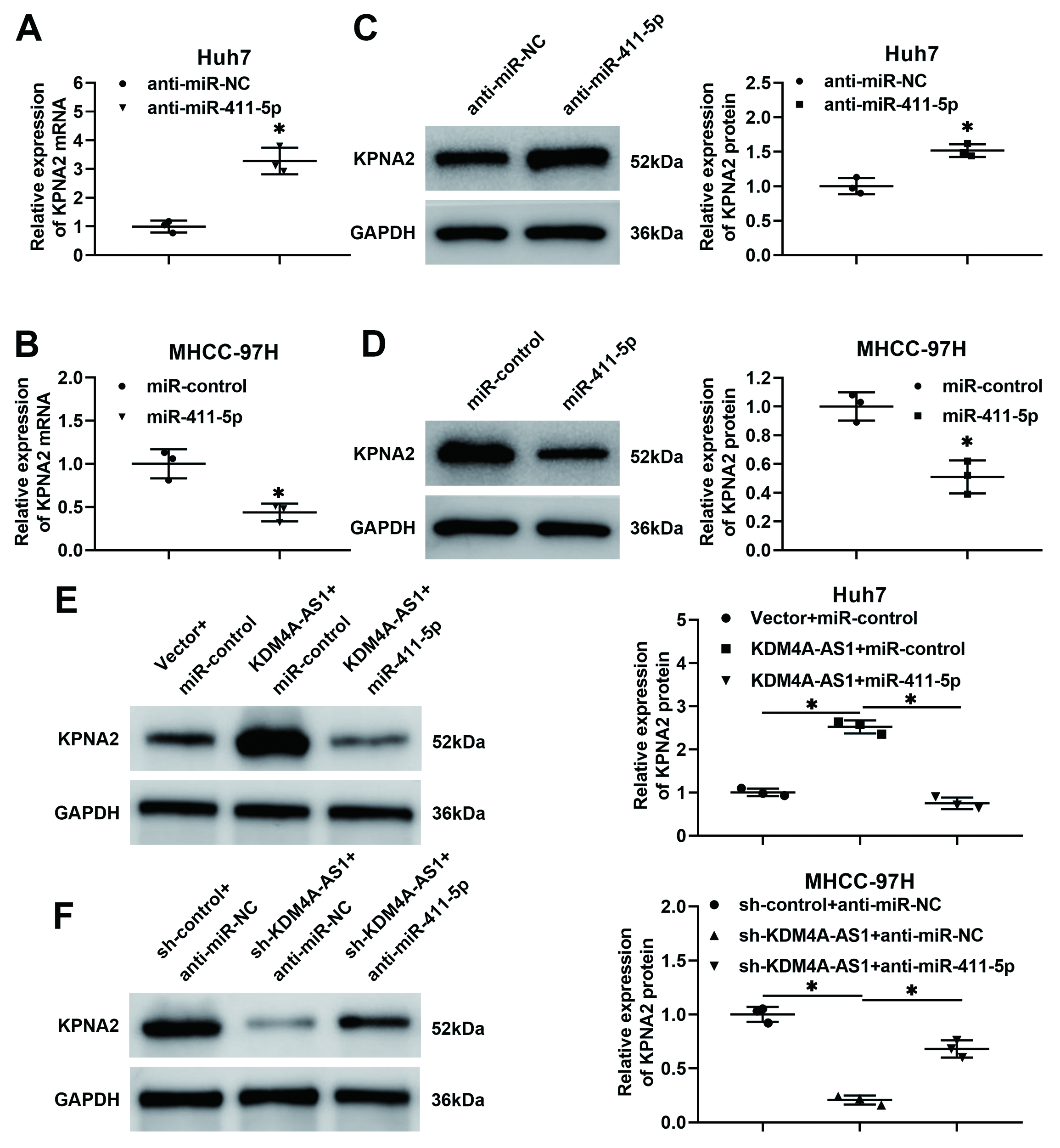

Supplement: Supplementary file 11 — Supplementary Figure 7 [file 41419_2021_4449_MOESM11_ESM.tif]

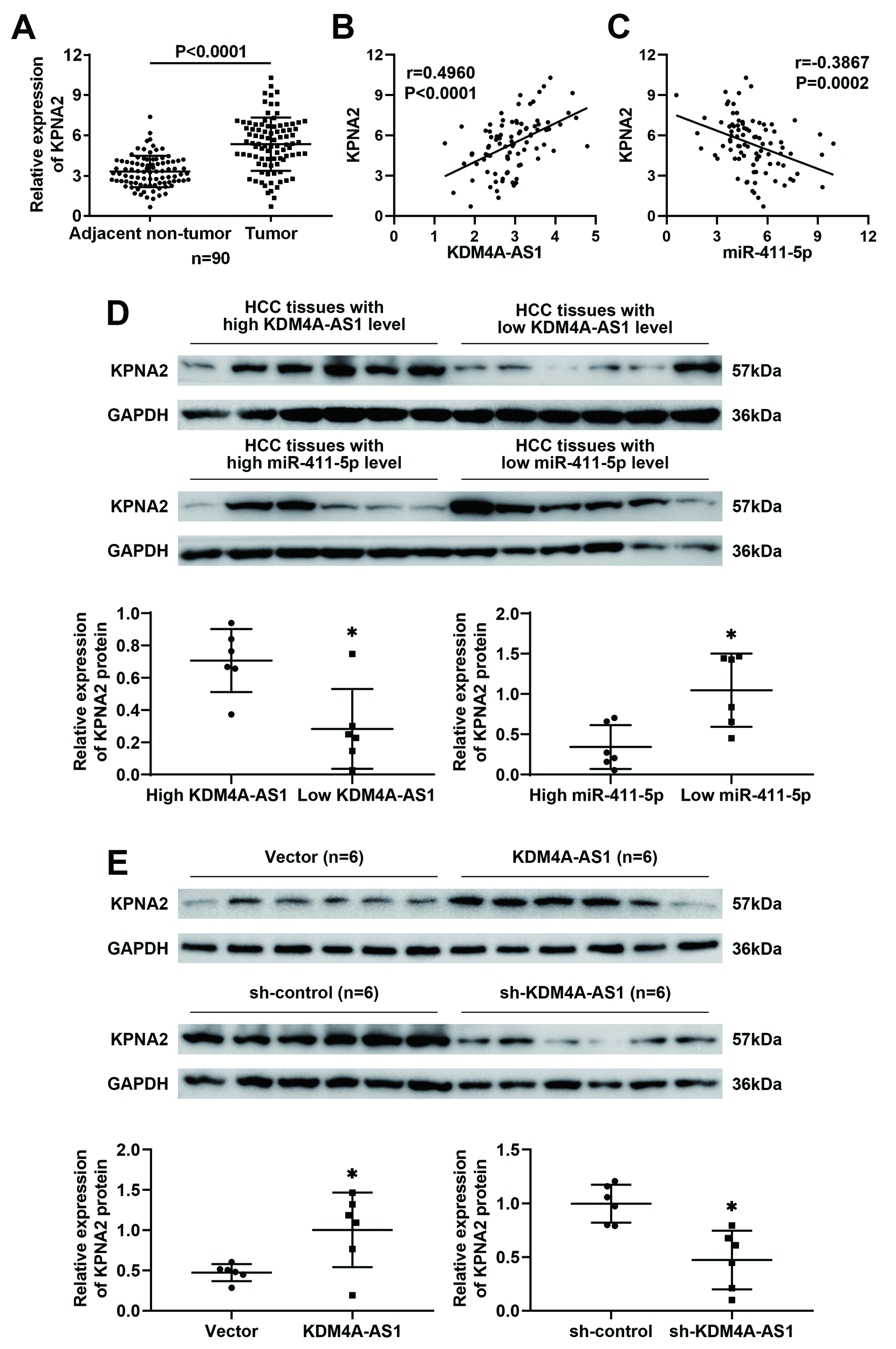

Supplement: Supplementary file 12 — Supplementary Figure 8 [file 41419_2021_4449_MOESM12_ESM.tif]

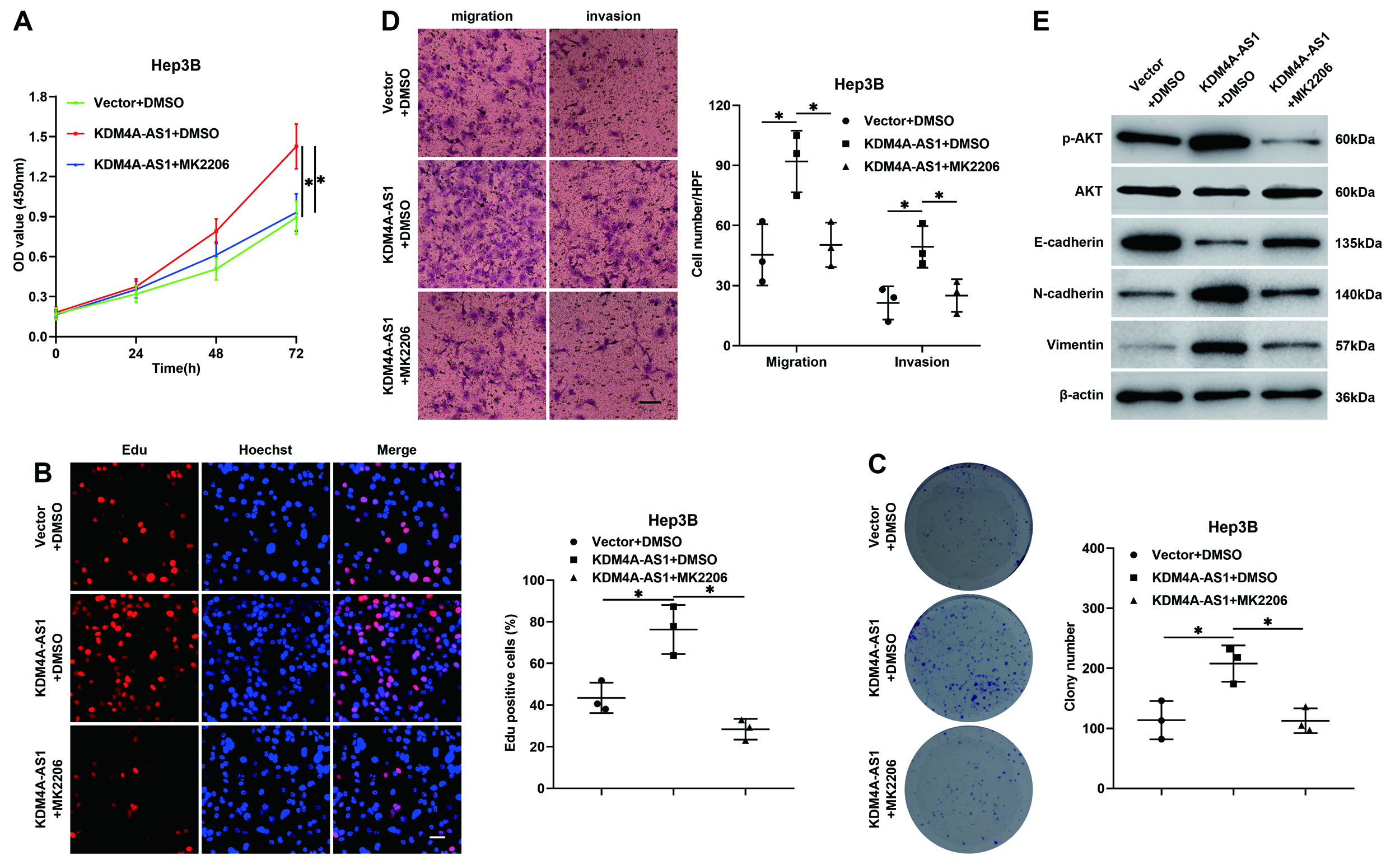

Supplement: Supplementary file 13 — Supplementary Figure 9 [file 41419_2021_4449_MOESM13_ESM.tif]
